# Assessing the revised Clinical Institute Withdrawal for Alcohol Scale use at Stikland Hospital

**DOI:** 10.4102/sajpsychiatry.v29i0.1915

**Published:** 2023-01-31

**Authors:** Creeshen P. Muddapah, Lize Weich

**Affiliations:** 1 Department of Psychiatry, Faculty of Medicine and Health Sciences, Stellenbosch University, Cape Town, South Africa

**Keywords:** alcohol withdrawal syndrome, alcohol use disorder, benzodiazepines, symptom-triggered detoxification, CIW-Ar, South Africa

## Abstract

**Background:**

Alcohol use disorder (AUD) is a major public health concern in South Africa (SA). Abrupt cessation or reduction of alcohol intake in chronic users can result in withdrawal symptoms. Benzodiazepines are the treatment of choice but need to be used cautiously in patients with a lifetime history of substance abuse given their highly addictive potential. Symptom-triggered prescription of benzodiazepines during alcohol withdrawal using the Revised Clinical Institute Withdrawal for Alcohol Scale (CIWA-Ar) has been associated with improved safety and reduced benzodiazepines use.

**Aim:**

To investigate if implementation of the CIWA-Ar during alcohol detoxification impacted the dose of benzodiazepines used and withdrawal-related outcomes.

**Setting:**

Alcohol rehabilitation unit (ARU) at Stikland Psychiatric Hospital.

**Methods:**

A retrospective cohort study of 135 admissions over a six-month period comparing two groups: before (2015) and after (2017) the implementation of the CIWA-Ar.

**Results:**

The study noted no differences in sociodemographic and alcohol-associated variables between the two groups, and there were no recorded complications in either group. The 2017 group had a lower percentage of patients that required benzodiazepines (33.8% vs. 51.4%, *p* = 0.04) and a lower median total amount of benzodiazepines used during alcohol withdrawal (0 mg vs. 5 mg, *p* = 0.01).

**Conclusions:**

The CIWA-Ar rating scale was an effective alternative to prescribing benzodiazepines pro re nata and decreased the total dose of benzodiazepines used during alcohol withdrawal.

**Contribution:**

The use of a symptom triggered regime, like the CIWA-Ar rating scale, during withdrawal can be implemented safely in a SA treatment setting for patients with low-risk AUD.

## Introduction

Alcohol is a psychoactive substance with dependence-producing properties,^1^ with alcohol use disorder (AUD) being the most prevalent mental health disorder and one of the leading risk factors for morbidity, disability and mortality in the world.^2^ The World Health Organization (WHO) 2011 Global Status Report ranked South Africa as a country with one of the riskiest patterns of alcohol consumption and with the highest reported alcohol consumption levels in Africa.^2^ A study from South Africa showed that alcohol use was associated with an increased risk of psychiatric disorders^3^ and has huge economic, social and health costs.^4^ In South Africa, the Western Cape has the highest prevalence of lifetime alcohol use (45%) and risky drinking behaviour (15%),^5^ where alcohol has been identified as the third most common primary substance of abuse for which treatment is sought, after methamphetamines and cannabis, respectively.^6^

Alcohol’s mechanism of action includes effects on multiple neurotransmitter systems and receptors, and its acute intake causes depression in the central nervous system (CNS) as a result of increased gamma-aminobutyric acid (GABA) neurotransmission and antagonist action at glutamate’s N-Methyl-D-Aspartate (NMDA) receptor.^7^ Chronic use of alcohol causes adaptive downregulation of GABA receptors and leads to an increased requirement of alcohol to produce the same effects, a phenomenon called tolerance.^8^ However, subsequent abrupt reduction or cessation of alcohol intake in the chronic user results in a reduction in the alcohol-mediated CNS inhibition and the glutamate-mediated CNS excitation being left unopposed, resulting in the clinical features of alcohol withdrawal.^8,9^ Alcohol withdrawal syndrome (AWS) starts within hours to a few days after cessation or reduction of heavy alcohol use and may present with: (1) sweating, (2) rapid pulse, (3) tremors, (4) insomnia, (5) nausea, (6) vomiting, (7) hallucinations, (8) agitation, (9) anxiety and (10) seizures.^10^ This disorder is potentially a life-threatening condition, especially in the case of complications including seizures or delirium tremens (DT).^9^

The effective management of AWS includes a combination of supportive and pharmacological measures that aims to make this period as comfortable and safe as possible.^11,12^ Benzodiazepines are currently recommended as the first drug of choice in the management of AWS^13^ but have substantial side effects in addition to being potentially highly addictive and therefore need to be used with caution in patients with a lifetime history of substance abuse.^14^ There are currently two regimens commonly used for prescribing benzodiazepines for alcohol withdrawal: the fixed-dose regimen (FDR) and the symptom-triggered (STR) regimens.^8^ The use of STR has been proven to effectively treat uncomplicated alcohol withdrawal and reduces the risk of over- and under-medication in patients.^15,16^ Previous studies have also found STR to be more advantageous compared with FDR because of shorter treatment duration, the lower total amount of benzodiazepines used (TABU) and reduced frequency of benzodiazepines used during the course of AWS in general medical settings and alcohol detoxification units.^15,16,17,18^

The Revised Clinical Institute Withdrawal Assessment for Alcohol Scale (CIWA-Ar) is a 10-item rating scale with a maximum score of 67, which assesses the clinically pertinent features of alcohol withdrawal.^19^ It is quick to administer, has high inter-rater reliability and allows for hourly repeated scoring. It is also not copyrighted and may be reproduced freely. The CIWA-Ar is a widely accepted and validated assessment tool used to monitor the severity of AWS and in titrating pharmacotherapy.^19^ The use of the CIWA-Ar rating scale during alcohol withdrawal was implemented at Stikland Hospital in 2016, and so the current study aims to evaluate its effects on the dose of benzodiazepines prescribed during alcohol detoxification and to assess its impact on withdrawal-related outcomes.

## Research methods and design

### Study design

This retrospective cohort study compared patients admitted to Ward 13, Stikland Hospital between January 2015 to June 2015 and January 2017 to June 2017, before and after the implementation of the symptom-triggered withdrawal protocol in 2016.

### Study setting

The study took place in Ward 13 at Stikland Hospital, a public psychiatric hospital in the Western Cape, South Africa. The unit houses a four week inpatient alcohol rehabilitation program, which is run by a multi-disciplinary team consisting of a psychiatrist, a medical officer, nurses, a psychologist, an occupational therapist and a social worker and offers an evidence-based group treatment program that focuses on addressing the patients’ substance use.

The program follows a self-referral route where patients are required to phone and book themselves into the program. After an initial telephonic screening by a nurse, patients are given an admission date according to a waiting list, from which around eight patients are admitted weekly. On admission, the patients are assessed by a medical officer, and if they are medically unstable or a complicated withdrawal is expected, they are redirected to a general medical hospital. Patients are required to stay on the ward premises full time and are usually allowed to go on leave from the second weekend.

Prior to the implementation of the CIWA-Ar linked STR for alcohol withdrawal in 2016, patients with AWS were given benzodiazepines, usually diazepam *pro re nata* at regular intervals according to the clinical judgement of the nursing staff. Since the implementation of the STR, the CIWA-Ar alcohol withdrawal scale is performed at regular intervals by nursing staff who received in-service training on administration of the CIWA-Ar scoring, and diazepam is prescribed according to the CIWA-Ar score per the alcohol rehabilitation unit’s (ARU) guidelines, where individuals scoring < 10 are considered to have mild withdrawal and not prescribed any diazepam and CIWA-Ar scoring done every 8 h until the score is < 6 on four successive occasions and the alcohol withdrawal is then considered completed. Individuals scoring 10–15 are considered to have moderate withdrawal and are prescribed 10 mg of diazepam orally, and individuals scoring > 15 are considered to have severe withdrawal and are prescribed 10 mg – 20 mg of diazepam orally, and CIWA-Ar scoring repeated hourly until the score is < 10 and then managed as above until alcohol withdrawal is considered completed.

### Study sample

The study population consisted of a randomly selected sample of 100 admissions (male and female) to Ward 13 at Stikland Hospital from 01 January 2015 to 30 June 2015 (153 admissions) and 01 January 2017 to 30 June 2017 (219 admissions), totalling 200 admissions for the study over the two 6-month periods. Thirty admissions from the 2015 group and 35 admissions from the 2017 groups were excluded from the study according to the exclusion criteria listed below, resulting in the final sample size of 135 patients (2015:70 and 2017:65) included in the study. Patients admitted multiple times during the above-mentioned time frames were counted as separate admissions.

#### General admission criteria to the programme

The alcohol rehabilitation programme admits any adult (≥ 18 years old) known with AUD and requires the patients to be motivated and to be able to participate in the voluntary programme for 4 weeks. There are no specific exclusion criteria to the unit, but patients noted to have active and unstable medical conditions or expected to have complicated withdrawal on admission are redirected towards a general medical hospital and not included in the programme and hence the study.

#### Exclusion criteria to the study

Patients with known allergies or sensitivity to benzodiazepines, active opioid or benzodiazepine use disorder, seizure disorders not related to alcohol withdrawal and last alcohol intake more than one week prior to admission.

### Data collection

Patients’ clinical files were retrieved from the Stikland Hospital Archives storage unit and collected by the principal investigator on a data collection sheet and collated in a Microsoft Excel spreadsheet. Data collected from patients’ medical files included sociodemographic details, admission details, alcohol use history, medical, psychiatric and substance use history and course and outcome of admission. Self-reported predominant pattern of drinking was also collected as follows: *daily or constantly* if patients drank on a daily or almost daily basis, as *4–6 days or week* if they drank at that frequency, *occasional* if they drank less than 4 days per week and *binges* if they drank ≥ 5 drinks (males) or ≥ 4 drinks (females) in about 2 h.^20^ When benzodiazepines other than diazepam were used, they were recorded in doses equivalent to 10 mg of diazepam according to a Benzodiazepine Equivalence Table.^21^

The outcome of the admission during the first 10 days was categorised as ‘uncomplicated withdrawal not requiring treatment’ if no treatment was required, ‘uncomplicated withdrawal requiring treatment’ if the patients received benzodiazepines without any complications, ‘complicated withdrawal’ if there were hallucinations, seizures or DT and ‘premature discontinuation’ if the patients left the programme for reasons other than complicated alcohol withdrawal, including early self-discharge during the first 10 days of admission.

### Data analysis

The collected data were analysed using SPSS Statistics, v27 software. Continuous variables were summarised as mean and standard deviation (s.d.) or median and interquartile range (IQR) based on normality. Nominal data were summarised as absolute counts and percentages. The *T*-test or Mann–Whitney test was used to determine statistical differences for continuous variables, and the chi-square test was used for nominal variables. The Kruskal–Wallis test was performed to assess the distribution of the TABU across the different predominant patterns of drinking. Statistical significance was set at *p* < 0.05 and derived from the above tests.

### Ethical considerations

Ethical approval was obtained from the Health Research Ethics Committee of Stellenbosch University (ref #: S20/12/350) and a waiver of consent was granted. Permission to access patient folders at Stikland Hospital was obtained from the South African National Department of Health and management of Stikland Hospital. The study was conducted in accordance with the South African Good Clinical Practice Guidelines (DOH 2006) and the Declaration of Helsinki (2013). All data were anonymised to ensure the privacy and confidentiality of participant’s personal information, with each participant assigned a unique identifier.

## Results

No differences in any of the sociodemographic variables between the two groups was noted (Table 1). The clinical characteristics were also comparable across the two groups except for medical comorbidities, where 67.1% of the 2015 group had at least one medical comorbidity compared with 43.1% in the 2017 group (*p* = 0.005). The medical comorbidities included: (1) hypertension (25.9%), (2) dyspepsia (18.5%), (3) previous tuberculosis (11.9%), (4) other chronic lung diseases (7.4%), (5) human immunodeficiency virus (HIV) (5.9%), (6) diabetes mellitus (3.0%), (7) dyslipidaemia (2.2%) and (8) other medical comorbidities (other gastrointestinal diseases, cardiovascular diseases, previous traumatic brain injuries, previous cerebrovascular accidents, dermatological and musculoskeletal diseases, 19.3%) across the two groups. Depressive disorders were found to be the most common psychiatric comorbidity (2015: 20%, 2017: 23.1%) and other comorbid substance use disorders were noted in approximately 15% of the study sample with cannabis use disorder being the most common at 14%.

**TABLE 1:** Sociodemographic and clinical characteristics of participants (*N* = 135).

Variable	2015 (*N* = 70)				2017 (*N* = 65)				Test value	*df*	*p*
	*N*	%	Mean	s.d.	*N*	%	Mean	s.d.			
**Sociodemographic characteristics**											
**Age**	-	-	41.74	9.52	-	-	42.66	9.85	−0.55	133	0.58
**Gender**									0.95	1	0.33
Male	44	62.9	-	-	46	70.8	-	-	-	-	-
Female	26	37.1	-	-	19	29.2	-	-	-	-	-
**Marital status**									3.70	2	0.16
Married	11	15.7	-	-	19	29.2	-	-	-	-	-
Single	55	78.6	-	-	42	64.6	-	-	-	-	-
Cohabiting	4	5.7	-	-	4	6.2	-	-	-	-	-
**Living situation**									0.18	1	0.67
Fixed	63	90	-	-	57	87.7	-	-	-	-	-
Homeless	7	10	-	-	8	12.3	-	-	-	-	-
**Clinical characteristics**											
**Medical comorbidities**									7.91	1	0.005
None	23	32.9	-	-	37	56.9	-	-	-	-	-
1	21	30.0	-	-	14	21.5	-	-	-	-	-
2	22	31.4	-	-	9	13.8	-	-	-	-	-
3 or more	4	5.7	-	-	5	7.7	-	-	-	-	-
**Psychiatric comorbidities** †									0.198	1	0.66
None	54	77.1	-	-	48	73.8	-	-	-	-	-
Depression	14	20.0	-	-	15	23.1	-	-	-	-	-
Other	5	5.7	-	-	4	6.0	-	-	-	-	-
**Other substance use** †									0.003	1	0.97
None	59	84.3	-	-	55	84.6	-	-	-	-	-
Cannabis	10	14.3	-	-	9	13.8	-	-	-	-	-
Other	4	5.8	-	-	3	4.6	-	-	-	-	-

s.d., standard deviation; df, degrees of freedom.

† , Total *n* for these variables was greater than the *n* of the group because of some participants having more than one psychiatric comorbidity and/or other substance use.

The alcohol-associated variables such as: (1) age at first alcohol intake, (2) duration of problematic drinking, (3) time since last alcohol intake at admission, (4) predominant pattern of drinking and (5) previous attendance to alcohol rehabilitation were similar across the two groups (Table 2). Moreover, approximately two-thirds of the patients reported drinking on a constant or daily basis (2015: 65.7%, 2017: 66.2%) compared with those who drank less frequently. More than a third reported having attended an alcohol rehabilitation programme previously (2015: 38.6%, 2017: 39.1%).

**TABLE 2:** Alcohol use history and course during admission of participants (*N* = 135).

Variable	2015 (*N* = 70)				2017 (*N* = 65)				Test value	*df*	*p*
	*n*	%	Median	IQR	*n*	%	Median	IQR			
**Alcohol use history**											
Age at first drink	-	-	18	5	-	-	17	3	0.228	130	0.20
Duration of problematic drinking (years)	-	-	11	17	-	-	11	16	−0.44	126	0.92
**Time since last alcohol intake**	-	-	-	-	-	-	-	-	2.41	2	0.30
< 24 h	39	55.7	-	-	42	64.6	-	-	-	-	-
24–72 h	23	32.9	-	-	20	30.8	-	-	-	-	-
72 h – 1 week	8	11.4	-	-	3	4.6	-	-	-	-	-
**Predominant pattern of drinking**	-	-	-	-	-	-	-	-	5.75	3	0.12
Constant	46	65.7	-	-	43	66.2	-	-	-	-	-
4–6 times per week	8	11.4	-	-	14	21.5	-	-	-	-	-
Occasional	3	4.3	-	-	0		-	-	-	-	-
Binges	13	18.6	-	-	8	12.3	-	-	-	-	-
**Previous attendance to alcohol rehabilitation**	27	38.6			25	39.1			0.03	1	0.96
**Withdrawal-related outcomes**											
Duration of stay	-	-	-	-	-	-	-	-	0.55	1	0.46
< 10 days	4	5.7	-	-	2	3.1	-	-	-	-	-
≥ 10 days	66	94.3	-	-	63	96.9	-	-	-	-	-
**Patients requiring benzodiazepine during alcohol withdrawal**	36	51.4			22	33.8			4.25	1	0.039
**Indication for benzodiazepine use**											
Alcohol withdrawal	36†	100	-	-	15‡	68.2	-	-	13.7	1	< 0.001*
High blood pressure	5†	13.9	-	-	13‡	59.1	-	-	14.2	1	< 0.001*
**TABU (mg of Diazepam)**	-	-	5	15	-	-	0	5	2.63	133	0.01
**Outcome of admission**	-	-	-	-	-	-	-	-	4.84	2	0.09
Uncomplicated without treatment	31	44.3	-	-	41	63.1	-	-	-	-	-
Uncomplicated with treatment	35	50.0	-	-	22	33.8	-	-	-	-	-
Premature discontinuation	4	5.7	-	-	2	3.1	-	-	-	-	-

IQR, interquartile range; df, degrees of freedom; TABU, total amount of benzodiazepines used.

* , *P*-values were calculated independently for alcohol withdrawal and high blood pressure; other indications such as agitation and others were not reported because of low numbers.

† , *N* = 36 and

‡ , *N* = 22.

Regarding withdrawal-related outcomes (Table 2), there was no statistical difference in the duration of stay (*p* = 0.46) and outcome of admission (*p* = 0.09) between the two groups, and no complicated alcohol withdrawals were reported in either group. The percentage of patients requiring benzodiazepines during alcohol withdrawal decreased from 51.4% in 2015 to 33.8% in 2017 (*p* = 0.039). Among those who received benzodiazepines in the 2015 cohort (*n* = 36), all 36 (100%) had alcohol withdrawal as an indication and five (13.9%) had high blood pressure also marked as an indication, compared with those in the 2017 cohort (*n* = 22) where 15 (68.2%) had alcohol withdrawal marked as an indication while 13 (59.1%) had high blood pressure marked as an indication. Notably, from the 2017 cohort, 20 patients (90.9%) received benzodiazepines even though their maximum CIWA-Ar scores were less than 10 and 17 patients (77.3%) had a maximum CIWA-Ar score of less than nine (data not shown). Furthermore, seven (31.8%) patients in the 2017 group received benzodiazepines with high blood pressure as the only indication, all with maximum CIWA-Ar scores of less than 10. The median (IQR) TABU decreased from 5 (15) mg in 2015 to 0 (5) mg in 2017 (*p* = 0.01), and it was also noted to be higher (*p* = 0.031) in those who reported drinking constantly compared with those who drank 4–6 times per week and less frequently (Table 3).

**TABLE 3:** Comparison of the total amount of benzodiazepines used of diazepam in all study participants across the predominant pattern of drinking (*N* = 135).

Predominant pattern of drinking	*N*	%	Median	Maximum	IQR	*p*
Constant	89	65.9	2.5	142.5	15	0.031
4–6 times per week	22	16.3	0	35	5	-
Mainly on weekends	21	15.6	0	115	0	-
Occasional binges only	3	2.2	0	0	0	-

IQR, interquartile range.

## Discussion

The purpose of the present study was to perform a chart review of admissions over a six month period before and after the implementation of the CIWA-Ar rating scale during alcohol withdrawal and to evaluate whether it reduced benzodiazepine use and improved withdrawal-related outcomes in patients admitted to an alcohol rehabilitation programme.

As evidenced by previous studies evaluating STR against FDR,^15,16,17,18^ the implementation of systematic and objective rating of withdrawal symptoms during alcohol withdrawal in the current study showed a statistically significant reduction in the TABU in the 2017 group when compared with benzodiazepines being prescribed *pro re nata* in the 2015 group. It was also consistent with a previous study in a naturalistic and low-risk population evaluating STR versus treatment as usual (TAU).^22^ However, the 2017 group also had a lower proportion of patients who reported having at least one medical comorbidity compared with the 2015 group, which might have been because of under-reporting from the patients or less strict documentation on admission. Notably, the patients in the current study all had stable chronic medical comorbidities, which are thought to contribute very little to the severity of AWS and are systematically underreported in this patient population.^23^ Both groups had similar drinking patterns and previous rehabilitation episodes; this supports the assumption that the groups had similar disease severity. The current study thus supports the evidence that using an STR protocol over *pro re nata* prescription is more advantageous in terms of lowering the TABU during alcohol withdrawal despite the increase in the most favourable outcome (alcohol withdrawal not requiring treatment) not attaining statistical significance.

Despite fewer patients in the post-CIWA-Ar implementation group requiring treatment during withdrawal, it was noted that among those who did receive benzodiazepines, the majority had their maximum CIWA-Ar score below the cutoff of 10. While some studies have recommended prescribing benzodiazepines during withdrawal even for CIWA-Ar scores between 8 and 10,^9,24,25^ it was also noted that almost a third of the patients received benzodiazepines for high blood pressure as the only indication with their CIWA-Ar scores being < 10. One explanation for the decreased prescription of benzodiazepines in the 2017 group could be that the nursing staff felt more comfortable with an objective measure of withdrawal symptoms and prescribed less benzodiazepines, with the notable exception of high blood pressure. The above implies that with stricter implementation of the CIWA-Ar during alcohol withdrawal and improved management of high blood pressure such as pre-admission screening and timely resumption of anti-hypertensives in those who had defaulted on their treatments, the TABU could have been minimised further.

There were no recorded complicated alcohol withdrawals across both groups, with most patients included in the programme completing at least 10 days post-admission. Only six patients prematurely discontinued the programme, with one patient from each group being diagnosed with active tuberculosis and being redirected to a specialised tuberculosis hospital while the remaining were self-discharges. The high completion rate of the programme and the absence of complicated alcohol withdrawals in the specialised rehabilitation programme are likely a result of the use of benzodiazepines during alcohol withdrawal, the enrolment of mostly low-risk patients through the self-booking process, thus implying higher motivation levels and the likelihood of patients lowering their alcohol consumption prior to admission, and the medical examination on admission where patients with unstable medical comorbidities are redirected to specialised medical facilities.

The majority of patients across both groups reported drinking daily or constantly compared with a less frequent predominant pattern of drinking, where severe alcohol dependence with higher frequency and higher daily units of alcohol consumption were associated with a higher risk of complications and higher amounts of benzodiazepine used during withdrawal.^8,26^ In the present study, patients who reported a higher frequency of alcohol consumption were noted to require more benzodiazepines during alcohol withdrawal across both groups, with the caveat that the data collected included only the self-reported predominant pattern of drinking without any specific timeline prior to admission and no quantification of their alcohol consumption. The above makes an argument for more detailed documentation about the pattern and amount of alcohol consumption in units per week in the weeks prior to admission or measurement of the blood alcohol levels upon admission to have better insight into the severity of alcohol dependence and its effects on alcohol withdrawal symptom severity. Furthermore, the current study also highlighted the high incidence of dual diagnosis and other comorbid substance use disorders among the patients, emphasising the need for screening and intervention for the above as part of holistic biopsychosocial rehabilitation in the target population.

The current study is the only one to our knowledge that evaluated the use of an objective withdrawal rating scale in a South African setting, where we showed that the use of the CIWA-Ar is a safe and effective alternative to the previously used *pro re nata* prescription of benzodiazepines, which relied heavily on the nursing staff’s level of experience with alcohol withdrawal. Furthermore, in comparison with the 2020 South Africa National Standard Treatment Guidelines for uncomplicated alcohol withdrawal at the primary health care level, which recommend FDR, totalling 100 mg of diazepam over a period of 1 week,^27^ the current study demonstrates that the implementation an objective withdrawal rating scale and STR during alcohol withdrawal could be beneficial in terms of reduction of benzodiazepine use while remaining safe and without any increase in negative outcomes if implemented at primary health care levels. The study also highlighted the need for stricter implementation of the CIWA-Ar scoring and optimisation of management of medical conditions during alcohol withdrawal to further limit the use of benzodiazepines and further supported the need for screening and intervention for other comorbid substance use disorders and dual diagnoses because of their high occurrence in the target population. However, there are a few potential limitations that need to be highlighted. First, it was a retrospective cohort analysis of standardised patient charts in one specialised ARU, which included a mostly low-risk and highly motivated population and excluded medically unstable patients, thus limiting its applicability to other general medical settings. Also, the current study did not evaluate other important factors in AUD rehabilitation like the patients’ comfort, satisfaction, duration of detoxification and relapse-associated factors in repeat admissions, which should be the focus of future studies.

## Conclusion

The present study demonstrated that the use of a rating scale linked STR during alcohol withdrawal in a specialised ARU is a safe and effective alternative and led to a decrease in the number of patients requiring benzodiazepines as well as a decrease in the TABU, without increasing the incidence of complicated alcohol withdrawals.
